# Examining the protective effects of caregiver-child closeness on the association between parenting behaviors and youth aggression

**DOI:** 10.1038/s41598-025-10151-6

**Published:** 2025-07-11

**Authors:** Elizabeth Kwon, Joon Jin Song, Yoo-Mi Chin, Aatiqah Hussain

**Affiliations:** 1 Department of Public Health, Baylor University, Rena Marrs McLean Gymnasium 233, One Bear Place #97343, Waco, TX 76798-7343 USA; 2 Department of Statistical Science, Baylor University, Waco, TX USA; 3 Department of Economics, Baylor University, Waco, TX USA

**Keywords:** Youth aggression, Parenting, Caregiver-child closeness, Emotional aggression, Physical aggression, Human behaviour, Risk factors

## Abstract

Youth aggression is a significant predictor of public health issues such as bullying, intimate partner violence, and homicide. This study investigates how caregiver-child closeness mitigates the association between parenting practices and youth aggression, using data from the Future of Families and Child Wellbeing Study (FFCWS). We first identified distinct dimensions of youth aggression and examined whether caregiver-child closeness could buffer the link between various parenting behaviors and different types of youth aggression. Two dimensions of youth aggression emerged in our dataset: emotional aggression and physical violence. The results show that non-violent discipline is positively associated with both types of aggression, but the strength of this association varies on the level of parental closeness. Specifically, when closeness is low, the links between non-violent discipline and both forms of aggression are stronger, whereas these associations are weaker when closeness is high. Notably, parental closeness did not significantly moderate the effects of psychological aggression, physical assault, or neglect. These findings suggest that while strengthening parent–child closeness may buffer the risks associated with non-violent discipline, it may be less effective in mitigating the impact of harsher parenting behaviors highlighting the need for tailored interventions based on specific parenting contexts.

## Introduction

Violence in all its forms has multifaceted and long-lasting impacts on the well-being of individuals and communities^1^. Globally, homicide, the most extreme form of violence, is the third leading cause of death in people aged 15–29 years^2^. With its epidemic-level prevalence, violence is deemed a public health issue where prevention is key^3^. While violence affects individuals across all age groups, special attention needs to be given to youth, given that this is a critical developmental stage that marks the onset of violent behaviors that can persist into adulthood^4^. Among the U.S. high school students who reported dating during the 12 months before the survey, 8.2% of youth participants reported experiencing physical dating violence, and 8.2% reported experiencing sexual dating violence based on the Youth Risk Behavior Survey data for 2019^5^. The same data showed that one in four high school students experienced bullying. Given its alarming level of prevalence and the urgent need for prevention, it is imperative to understand the risk and protective factors of youth violence. The current study aims to understand youth aggression, a precursor of more severe violence later, in the context of family dynamics. Given the interconnection between family and youth violence^1^ and the importance of the parent-child relationship as a violence prevention mechanism^6^, a deeper understanding of the association between family context and youth aggression will help identify specific familial risk and protective factors for aggressive behaviors.

### Youth aggression as a precursor of adult violence

Youth aggression is a well-established predictor of broader public health concerns, particularly violence. The life-course-persistent offending theory^7^ suggests that aggressive behaviors at early ages are strongly predictive of adverse adulthood behavioral outcomes such as violent crimes against women and children. Further, self-reported aggression in adolescent offenders was found to be a strong predictor of future arrests for violent offenses as well as delinquency^8^. These findings highlight the importance of identifying and addressing aggression at early ages. Given the developmental consequences of aggression at early ages, it is essential to understand how aggression is formed among younger populations and to develop interventions that can mitigate the long-term effects of aggression on individuals and society.

### Parental behaviors as a major predictor of youth aggression

One major contributing factor to youth aggression is parental behaviors. As the social learning theory suggests, youth aggression can result from observational learning where children observe violent interactions among adults and imitate them^9^. For example, witnessing interparental violence increases the risk of youth perpetration of dating violence^10,11^. The mechanism of the association is that children exposed to violence in their household may perceive violence and aggression as an acceptable option for conflict resolution or as a common aspect of intimate relationships^12,13^. Further, negative parenting practices can contribute to youth aggression. Specifically, corporal punishment (e.g., spanking, slapping)^14^, controlling and authoritarian parenting styles^15^, and psychological control^16,17^ have been found to raise the levels of youth aggression. The association between negative parenting practices and youth aggression is mediated by traits such as youth’s endorsement of violence, negative emotions, and low self-control^14,18^.

As much as parents’ negative behaviors can elevate youth aggression, positive parental behaviors can alleviate aggressive tendencies among youth. It is well established that the quality of parent-child attachment has a significant impact on youth development by shaping emotional security and self-regulation, thereby reducing the likelihood of aggressive behaviors^19–23^. In particular, parental closeness, defined as feelings of connectedness, affection, and warmth between a parent and their child^24^, is associated with lower levels of aggressive behaviors among youth^25,26^. The positive impact of parental closeness on youth behavior is found to be greater for younger adolescents than for older adolescents^24^. These findings suggest that interventions aimed at strengthening parent-child relationships, particularly during early adolescence, could be key to mitigating youth aggression.

### Parental closeness as a moderator of parenting behaviors

Parental closeness may interact with parenting style in shaping youth behavior. Some studies found that parental warmth moderates the negative effects of harsh parenting on youth’s externalizing problems^27^. Similarly, several studies showed that maternal warmth can buffer even the negative effects of corporal punishment^28,29^. On the other hand, other studies found that harsh parenting and punishment directly increased youth problem behaviors, irrespective of parental closeness^30,31^. Therefore, whether and to what extent parental closeness can protect youth from the effects of negative parenting behaviors remains inconclusive.

### Differential impact of parenting by type of aggression

Moreover, the buffering effects of parental closeness may be conditional on specific parenting practices or youth aggression types. Youth physical aggression, characterized by overt violence, may be less influenced by parental closeness in the presence of corporal discipline because physical punishment directly models and reinforces overt violent behavior, which may override the potential protective effects of warmth. On the other hand, relational aggression, a covert form of aggression characterized by manipulative behaviors (e.g., intentionally excluding someone from a group activity, spreading malicious rumors or gossip, threatening to end a relationship) may be more sensitive to the effects of parental closeness. When children experience warm and secure relationships with their parents, they may not feel the need to use manipulative tactics in their relationships with other people. Indeed, Kuppens et al.^32^ found that parental corporal punishment was associated with child’s overt aggression, while parental psychological control was associated with relational aggression, with no evidence of cross-over effect. Another study found that while the authoritarian parenting style, which is characterized by a lack of parental warmth, did not increase physical aggression, it was associated with higher levels of relational aggression among children^33^. Research has consistently demonstrated that various dimensions of aggression have distinct antecedents and consequences^34–36^. Understanding the differential effects of parenting on distinct types of aggression is crucial as it informs tailored interventions. Parsing out the specifications of links will inform more precise prevention strategies.

Despite this need, the existing literature is limited in that the moderating effect of parental closeness has not been tested in varied contexts with different parenting practices and distinct types of aggression. To capture the complex dynamics among parenting, caregiver-child closeness, and youth aggression, it is crucial to analyze the combined impact of parenting and closeness on different types of aggression. The current study addresses this gap. The current study evaluates the extent to which parental closeness mitigates the association between negative parenting and youth aggression, specifically by different types of aggression. We identify distinct types of aggression within our data by conducting an exploratory factor analysis. With these identified types of aggression, we investigate whether the association between parenting behaviors and distinct types of aggression varies depending on the levels of parental closeness.

## Methods

### Data and sample

Secondary data analyses were conducted using the Future of Families and Child Wellbeing Study (FFCWS) study. The FFCWS is a longitudinal birth-cohort study following 4,898 children and their caregivers in 20 large U.S. cities with a population of 200,000 or more. Sampling was conducted first by cities, then by hospitals within cities, and finally, by births within hospitals^37^. The FFCWS oversampled unmarried mothers by a ratio of 3 to 1 to ensure inclusion of low-income families. Baseline data (wave 1) was collected between 1998 and 2000 when children were born, and participants were followed up when children were ages 1 (wave 2), 3 (wave 3), 5 (wave 4), 9 (wave 5), and age 15 (wave 6). The current study conducted a cross-sectional analysis using wave 5 data (collected between 2007 and 2010) to focus on youth aggression at a specific developmental stage (age 9) when early aggressive behavior patterns become more evident. The FFCWS data were collected with informed consent, and both participant consent and assent were obtained. These procedures were overseen by Princeton University’s Institutional Review Board. As shown in Table 1, the final sample included 2,297 youth with a mean age of 9.2 (standard deviation [SD] = 4.07). Male youth consisted 51% of the sample, and the majority were non-Hispanic Black (51.2%) followed by Hispanic (22.8%). The average poverty level was 2.30 (SD = 2.30) indicating household income was 2.3 times the poverty level.

**Table 1:** Sample characteristics.

	Mean/frequency	SD/%
Age	110.782	4.07
Sex (male)	1177	51.1%
Race		
White only, non-Hispanic	424	18.3%
Black/Af. American only, non-Hispanic	1178	51.2%
Hispanic/Latino	526	22.8%
Other only, non-Hispanic	64	2.7%
Multi-racial, non-Hispanic	120	5.1%
Poverty ratio	2.304	2.30
Primary caregiver		
Biological mother	2217	96.4%
Biological father	86	3.6%
Parenting		
Non-violent discipline	9.187	6.01
Psychological aggression	3.407	3.39
Physical assault	1.313	2.17
Neglect	0.218	0.75
Closeness	3.591	0.52
Overall aggression at W4	8.68	5.90
Parenting stress	8.06	2.67
Aggression		
Emotional aggression	-0.014	0.93
Physical violence	-0.018	0.90

Listed variables are from W5 except for the child’s sex and overall aggression. Age is in months. Poverty ratio is the ratio of the primary caregiver’s household income/poverty threshold. *SD * standard deviation.

### Measures

Parenting behavior was measured using the Conflict Tactics scale^38^, which consists of 4 subscales, including non-violent discipline, psychological aggression, physical assault, and neglect. Primary caregivers were asked to report how often in the past year they were involved in each of the 19 parenting behaviors. Response options included categories like ‘never,’ ‘once,’ ‘3–5 times,’ ‘11–20 times,’ and ‘more than 20 times.’ These categorical responses were then converted to counts by using the midpoint of each range, with the highest category (‘more than 20 times’) being assigned a value of 21. Non-violent discipline was measured using 4 questions including “parent has explained why something child did was wrong” and “parent has taken away privileges” (alpha = 0.84). Psychological aggression was measured using 5 questions (e.g., parent has shouted, yelled or screamed at child; parent has sworn or cursed at child, alpha = 0.64). Physical assault was measured using 5 questions (e.g., parent has slapped child on hand, arm or leg, alpha = 0.72). Neglect was assessed using 5 questions (e.g., parent not able to make sure child got food needed, alpha = 0.58). For each subscale, the average score was calculated.

Aggression was measured using the Child Behavior Checklist (CBCL)^39^. A parent or caregiver rated their child’s aggressiveness on 18 items (e.g., child argues a lot, gets in many fights, sulks a lot, threatens people, physically attacks people) from 1 (Not true) to 3 (Very true or often true), with higher scores indicating greater levels of aggression. These 18 items were used to conduct the Exploratory Factor Analysis.

Caregiver-child closeness was measured based on the caregiver report on two questions: how close they feel with the child (1 = not very close, 4 = extremely close); and how well the child shares ideas or talks with the caregiver (1 = not very well, 4 = extremely well). The average of the two scores was calculated to represent overall caregiver-child closeness.

Control variables included age, biological sex, race/ethnicity (White only, non-Hispanic; Black/African American only, non-Hispanic; Hispanic/Latino; Other only, non-Hispanic; Multi-racial, non-Hispanic), poverty (primary caregiver’s household income/poverty threshold; higher value indicates more affluent family environment), and primary caregiver. Overall aggression at wave 4 was measured by the sum score of the 18 items of the CBCL aggression subscale. Parenting stress was measured by the sum score of the 4 items derived from the Child Development Supplement of the Panel Study of Income Dynamics.

### Statistical analysis

The Exploratory Factor Analysis (EFA) was conducted to find the underlying structure of the aggression measure following previous research^40^. Eigenvalues suggested a 2-factor latent variable: emotional aggression and physical violence. Of the total 18 items of aggression, 10 items loaded on the emotional aggression factor, with factor loadings ranging from − 0.882 to 4.765. Example items included ‘child is stubborn, sullen, or irritable,’ ‘child has temper tantrums or a hot temper,’ ‘child has sudden changes in mood or feeling,’ ‘child argues a lot.’ Seven items were loaded on the physical violence factor with factor loadings ranging from − 0.561 to 6.669. Items included ‘child physically attacks people,’ ‘child gets in many fights,’ ‘child destroys things belonging to family or others,’ and ‘child is cruel, bullies, or shows meanness to others.’ The Kaiser-Meyer-Olkin (KMO) measure of sampling adequacy was 0.92, and Bartlett’s test of sphericity was significant (*p* < .001), confirming the suitability of the data for factor analysis. To explore whether caregiver-child closeness influences the association between parenting behaviors and aggression, we examined its moderating effects. Two regression models were conducted—one for each aggression outcome. Interaction effects were tested simultaneously by including four interaction terms, each representing the interaction between closeness and one of the four parenting behaviors. Significant interactions were further examined using simple slopes plots. Analyses were conducted with complete cases. All statistical analyses were conducted using R software.

## Results

As shown in Table 2, older age was associated with higher levels of emotional aggression but not with physical violence. Female sex was not significantly associated with emotional aggression but was associated with lower levels of physical violence. Black children were less likely to show emotional aggression compared to White, non-Hispanic children. Race/ethnicity was not significantly associated with physical violence. A higher poverty ratio, which indicates higher household income, was associated with lower physical violence and emotional aggression.

**Table 2:** Regression models for physical violence and emotional aggression.

	Physical violence				Emotional aggression			
	Beta	SE	95% CI		Beta	SE	95% CI	
Age	0.030	0.018	-0.007	0.066	0.050**	0.017	0.016	0.084
Sex (ref. male)								
Female	-0.073**	0.018	-0.109	-0.036	-0.028	0.017	-0.061	0.006
Race (ref. white, non-Hispanic)								
Black/Af. American only, non-Hispanic	0.012	0.027	-0.042	0.065	-0.062*	0.026	-0.112	-0.012
Hispanic/Latino	-0.022	0.026	-0.072	0.029	-0.032	0.024	-0.079	0.015
Other only, non-Hispanic	-0.012	0.019	-0.050	0.026	-0.012	0.018	-0.048	0.023
Multi-racial, non-Hispanic	0.003	0.020	-0.037	0.043	-0.004	0.019	-0.041	0.034
Poverty ratio	-0.057*	0.020	-0.096	-0.018	-0.053**	0.019	-0.090	-0.017
Primary caregiver (ref. biological mother)								
Biological father	0.026	0.018	-0.010	0.062	0.014	0.017	-0.019	0.048
Parenting								
Non-violent discipline	0.457**	0.153	0.158	0.756	0.595**	0.142	0.316	0.875
Psychological aggression	0.231	0.170	-0.102	0.564	-0.046	0.159	-0.357	0.265
Physical assault	0.169	0.135	-0.095	0.434	0.213	0.126	-0.034	0.460
Neglect	-0.010	0.114	-0.234	0.214	0.101	0.107	-0.109	0.310
Closeness	-0.011	0.033	-0.053	0.076	-0.009	0.031	-0.069	0.051
Overall aggression at W4	0.289**	0.019	0.251	0.327	0.341**	0.018	0.306	0.377
Parenting stress	0.059**	0.019	0.021	0.097	0.103**	0.018	0.068	0.138
Interaction terms								
Non-violent discipline * Closeness	-0.347*	0.154	-0.649	-0.046	-0.477**	0.144	-0.729	-0.165
Psychological aggression * Closeness	-0.134	0.168	-0.464	0.196	0.183	0.157	-0.125	0.491
Physical assault * Closeness	-0.118	0.134	-0.382	-0.145	-0.175	0.125	-0.421	0.071
Neglect * Closeness	0.065	0.113	-0.157	0.228	-0.037	0.106	-0.245	0.171

Listed variables are from W5 except the child’s sex and overall aggression. Poverty ratio is the ratio of the primary caregiver’s household income/poverty threshold. beta , standardized regression coefficient beta; SE, standard error; CI, confidence interval; ref, reference group. *p < 0.05. **p < 0.01.

**Fig. 1:**
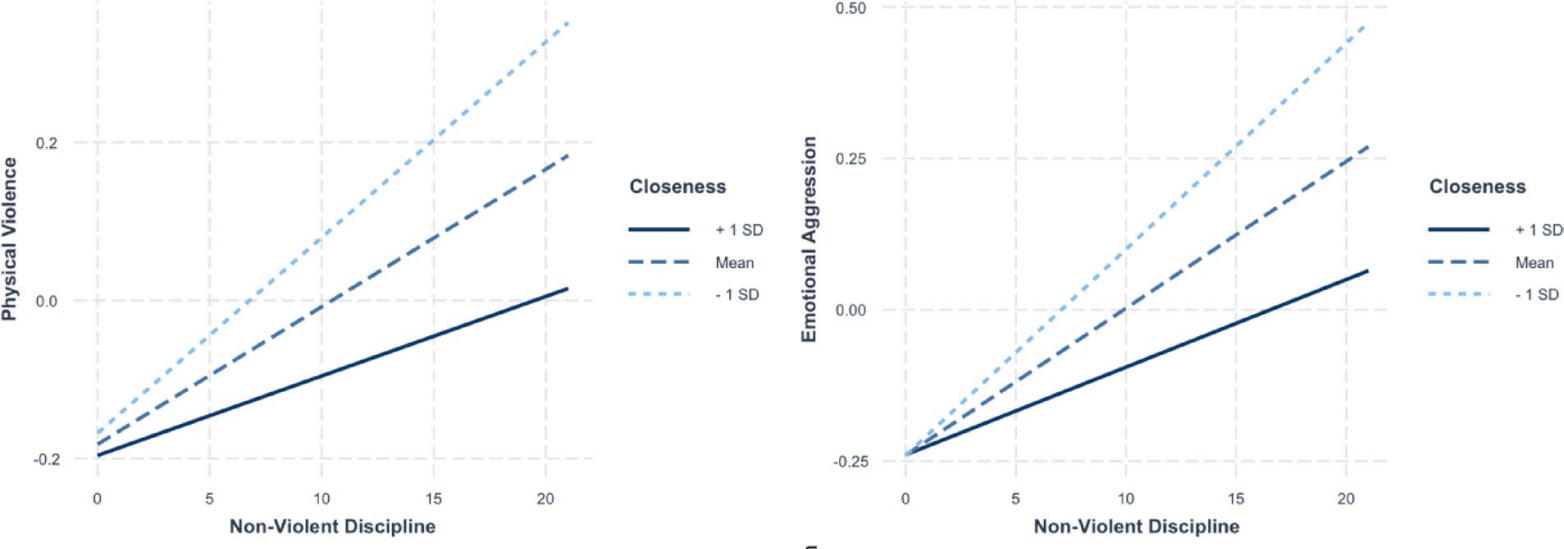
Simple slopes plots depicting the moderating effect of parental closeness on the association between non-violent discipline and two forms of aggression: physical violence (left panel) and emotional aggression (right panel).

Table 2 shows that non-violent discipline was directly associated with both physical violence and emotional aggression, even when controlling for other parenting behaviors. Moreover, parental closeness significantly moderated these associations. As illustrated in Fig. 1, the relationships between non-violent discipline and both forms of youth aggression were stronger when parental closeness was low (− 1 standard deviation [SD]). Conversely, when parental closeness was high (+ 1 SD), these associations were attenuated, indicating that high closeness buffers or weakens the link between non-violent discipline and children’s aggressive behaviors. This pattern was consistent across both aggression types.

## Discussion

The current study examined the role of parental closeness in mitigating the association between parenting behaviors and youth aggression. Our first goal was to categorize a broad range of child aggressive acts within the data into several distinct types of aggression. Conducting factor analysis on 18 items, we identified two distinct aggression types: *physical violence* and *emotional aggression*. *Physical violence* is characterized by overt behavioral expressions of anger such as hitting, pushing, or shoving^33^, and *emotional aggression* is characterized by irritable temperament, difficulty in emotion regulation, and argumentative traits. This finding is consistent with a previous study that found two distinct factors of aggression using CBCL items and the same data (FFCWS)^40^.

Our study is well situated within the aggression literature, which frequently discusses distinct dimensions of aggression. While the majority of aggression-related research has focused on physical aggression, increasing attention has been given to relational aggression - manipulative forms of aggression intended to harm social relationships - since Crick and Grotpeter^41^ first introduced this concept. Since then, researchers have investigated physical and relational aggression as separate constructs and empirical evidence supported the distinction by demonstrating unique antecedents and consequences between the two^34,35^. Our study contributes to the literature by introducing *emotional aggression*, a unique dimension distinct from physical or relational aggression. Although we could not measure relational aggression due to the lack of the relevant variables in the FFCWS data, we identified this distinct factor representing emotional traits such as irritability, hot temper, sudden mood change, and sulking. This new dimension is different from physical or relational aggression in that it is more emotional and dispositional rather than behavioral.

Further, we found distinct correlates for different types of aggression, consistent with prior research identifying unique antecedents of relational versus physical aggression^36^. Female sex was associated with lower levels of physical violence. In contrast, emotional aggression was positively associated with older age and negatively associated with non-Hispanic Black race. This finding highlights that different forms of aggression may have unique developmental and cultural influences, underscoring the importance of tailoring prevention and intervention efforts to address specific types of aggression within diverse populations.

The moderating effect of closeness did not differ by aggression type. Parental closeness significantly moderated the association between non-violent discipline and both forms of aggression, controlling for other interaction terms. Notably, no significant moderating effects were found for the interactions between closeness and other parenting behaviors, highlighting that parental closeness uniquely buffers the impact of non-violent discipline but not other parenting practices. This specificity suggests that the protective role of closeness may depend on the type of parenting behavior involved. The consistent moderating effect of closeness on non-violent discipline across aggression types underscores the importance of fostering parental closeness as a protective factor within the context of non-violent discipline. However, it also suggests that interventions may need to target other parenting behaviors differently. Indeed, previous studies found that certain forms of parenting behaviors (e.g., psychological aggression) may have stronger effects on youth development than other forms^42,43^. Future research should investigate why closeness fails to moderate harsher or more harmful parenting behaviors and consider how different aspects of the parent-child relationship may influence these dynamics.

The current study has limitations. First, our analysis relied on parent-reported measures which may be subject to social desirability bias. This could have led parents to underreport undesirable parenting behaviors, attenuating the associations found in our analyses and resulting in an underestimation of the relationships. Second, we focused on age 9 data conducting cross-sectional analyses due to inconsistency in variable availabilities across different waves of the FFCWS data. This limits our ability to draw conclusive causal relationships. Given the wealth of research indicating a bidirectional association between parenting and child behavior^44^, future research should address these limitations by integrating longitudinal study designs. Additionally, wave 5 of the FFCWS was collected between 2007 and 2010. Although the data are not recent, we believe the underlying mechanisms examined are stable and unlikely to be highly sensitive to the period of data collection. Nevertheless, future research should replicate these findings using more recent data to confirm their continued relevance.

The current study produced two important findings. First, we identified a unique subcategory of aggression: emotional aggression. This form of aggression, distinct from physical violence or relational aggression, is characterized by irritability, mood instability, and argumentative behaviors. Second, we found that parental closeness does not buffer the association between parental psychological aggression, physical assault, and neglect and youth emotional aggression. These findings carry several important implications. First, there is a need to further refine how aggression is conceptualized to develop more targeted and effective interventions. Second, the harmful effects of parental psychological aggression, physical assault, and neglect on youth aggression may not be adequately offset by parental closeness, highlighting the necessity of prioritizing prevention efforts. Finally, since fostering closeness alone may be insufficient, especially in families experiencing abuse and neglect, it is critical to consider the broader family context and the quality of parent-child interactions when designing and implementing interventions.

## Data Availability

The data that support the findings of this study are publicly available from https://ffcws.princeton.edu/.
